# Development of fluorescence quenching in *Chlamydomonas reinhardtii* upon prolonged illumination at 77 K

**DOI:** 10.1007/s11120-018-0534-8

**Published:** 2018-06-13

**Authors:** Lucyna M. Wlodarczyk, Joris J. Snellenburg, Jan P. Dekker, Ivo H. M. Stokkum

**Affiliations:** LaserLaB, Department of Physics and Astronomy, Faculty of Science, Vrije Universiteit Amsterdam, De Boelelaan 1081, 1081 HV Amsterdam, The Netherlands

**Keywords:** Photosystem II, State transitions, Time-resolved fluorescence, Target analysis

## Abstract

Low-temperature fluorescence measurements are frequently used in photosynthesis research to assess photosynthetic processes. Upon illumination of photosystem II (PSII) frozen to 77 K, fluorescence quenching is observed. In this work, we studied the light-induced quenching in intact cells of *Chlamydomonas reinhardtii* at 77 K using time-resolved fluorescence spectroscopy with a streak camera setup. In agreement with previous studies, global analysis of the data shows that prolonged illumination of the sample affects the nanosecond decay component of the PSII emission. Using target analysis, we resolved the quenching on the PSII-684 compartment which describes bulk chlorophyll molecules of the PSII core antenna. Further, we quantified the quenching rate constant and observed that as the illumination proceeds the accumulation of the quencher leads to a speed up of the fluorescence decay of the PSII-684 compartment as the decay rate constant increases from about 3 to 4 ns^− 1^. The quenching on PSII-684 leads to indirect quenching of the compartments PSII-690 and PSII-695 which represent the red chlorophyll of the PSII core. These results explain past and current observations of light-induced quenching in 77 K steady-state and time-resolved fluorescence spectra.

**Electronic supplementary material:**

The online version of this article (10.1007/s11120-018-0534-8) contains supplementary material, which is available to authorized users.

## Introduction

Photosynthesis starts with the absorption of a photon by a light-sensitive molecule, typically chlorophyll. What happens next has been extensively studied by means of fluorescence spectroscopy already since the XIXth century (Müller 1874; Govindjee 2004). In the late 1950s, these studies were extended to cryogenic temperatures (Brody 1958). The resulting higher spectral resolution revealed the existence of emission bands which were not detected in photosynthetic samples at ambient temperatures and which originated from Photosystem I (PSI) (Brody 1958) and Photosystem II (PSII) (Litvin et al. 1960; Govindjee and Yang 1966), two major photosynthetic pigment–protein complexes that convert light energy into electrochemical energy. This increased spectral resolution came however with a drawback. In their study on whole leaves and isolated chloroplasts from spinach, Kyle et al. (1983) observed that light-induced quenching of fluorescence occurs at 77 K which is especially strong in PSII. Several further works confirmed that this finding also in PSII cores isolated from cyanobacteria (Schweitzer et al. 1998), PSII particles from pea (Siffel et al. 2000), PSII membranes from spinach (Schweitzer and Brudvig 1997; Schweitzer et al. 1998; Mohamed et al. 2016), pea (Siffel et al. 2000) and *Chlamydomonas reinhardtii* (Wang et al. 2002) and in leaves of pea (Siffel et al. 2000). The wealth of different techniques used in these studies has provided insight into this light-induced 77 K quenching in the PSII core.

Under physiological conditions, the excitation energy leads to a charge separation between a chlorophyll molecule, the PSII primary donor P680, and a pheophytin molecule, Pheo. The ejected electron is rapidly transferred to the electron acceptor plastoquinone *Q*_A_ and then to plastoquinone *Q*_B_. The primary electron-transfer pathway is completed when the oxidized P680 (P680^+^) is reduced by the S-state cycle via the redox-active tyrosine residue *Y*_Z_ (Schweitzer and Brudvig 1997). At cryogenic temperatures, however the electron-transfer pathway changes (De Paula et al. 1985). It has been suggested that because below 100 K the electron needed to reduce P680^+^ is not derived from the S-state cycle, it is provided instead by Chl_Z_ (De Paula et al. 1985; Schweitzer and Brudvig 1995, 1997). In most of the studies on the light-induced fluorescence quenching at cryogenic temperatures, this oxidized Chl_Z_ molecule is indicated as the quencher; however, also other molecules have been considered (Okayama and Butler 1972; Kyle et al. 1983; Schweitzer and Brudvig 1997). Schweitzer and Brudvig (1997) demonstrated that if 15% of Chl_Z_ molecules present in the photosynthetic apparatus are oxidized, they quench 70% of the low-temperature fluorescence. This finding indicates that the PSII complexes embedded in photosynthetic membranes are energetically connected. In another study, the authors concluded that if a photosynthetic sample at cryogenic temperature is illuminated it is difficult to avoid creating the quenching effect (Schweitzer et al. 1998).

Nearly 70 years after the first experiments, low-temperature fluorescence spectra are still widely used as a reference to study the distribution of absorbed excitation energy between PSI and PSII. A primary example of a mechanism that regulates this distribution in higher plants, algae and cyanobacteria are state transitions (ST) (van Thor et al. 1998; Allen 2003; Bellafiore et al. 2005; Drop et al. 2014), for the first time observed in 1969 in 77 K fluorescence spectra of *Porphyridium cruentum* (Murata 1969). Many subsequent studies on ST have routinely included low-temperature fluorescence spectroscopy (Allen et al. 1981; Wollman and Delepelaire 1984; Bruce et al. 1985; Delphin et al. 1995; Cardol et al. 2003; Bellafiore et al. 2005; Kargul et al. 2005; Chuartzman et al. 2008; Iwai et al. 2008; Allorent et al. 2013; Drop et al. 2014; Ünlü et al. 2016). It is commonly accepted that redistribution of excitation energy between PSI and PSII upon state transitions is realized by a shift of a part of the light-harvesting antenna of PSII (LHCII) between PSII (state 1; St1) and PSI (state 2; St2). In the green algal model organism *Chlamydomonas reinhardtii* (*C. reinhardtii*), the reported amount of LHCII involved in ST (so-called mobile LHCII) varies greatly between studies and there is so far no conclusion on how much of LHCII detached from PSII re-attaches to PSI (Wollman and Delepelaire 1984; Delepelaire and Wollman 1985; Delosme et al. 1996; Nagy et al. 2014; Ünlü et al. 2014; Wlodarczyk et al. 2015; Nawrocki et al. 2016; Snellenburg et al. 2017). It is therefore important to account for possible biases that might be inherent to the techniques applied in these studies. Since light-induced fluorescence quenching at 77 K affects PSI and PSII differently, it should be carefully evaluated with respect to its influence on ST estimation based on low-temperature fluorescence spectra. On the other hand, quenching can serve as a probe to study the functional connectivity between photosynthetic complexes (Schweitzer et al. 1998). This approach is interesting with regard to the state transitions which involve remodelling of the photosynthetic apparatus.

In our previous work, we have developed a unified model for the excited state dynamics in the photosynthetic apparatus of intact cells of *Chlamydomonas reinhardtii* frozen to 77 K (Wlodarczyk et al. 2015; Snellenburg et al. 2017). Simultaneous analysis of the time-resolved fluorescence spectra measured in *C. reinhardtii* WT and photosynthetic mutants under state 1 and state 2 conditions allowed us to effectively resolve the internal dynamics of the LHCII–PSII complex as well as the PSI complex with its light-harvesting antenna (LHCI) and LHCII antenna (LHCII–LHCI–PSI). In this work, accumulation of the low-temperature quenching induced upon prolonged illumination was studied in intact cells of the model organism *Chlamydomonas reinhardtii*. We used the target model to analyse the measured series of time-resolved fluorescence spectra and we demonstrate how this light-induced 77 K fluorescence quenching is resolved by the model.

## Materials and methods

### Sample

*Chlamydomonas reinhardtii* wild-type (WT) strain 137c was a kind gift of Prof. Jean-David Rochaix. The cells were grown under 25 µmol photons PAR m^− 2^s^− 1^ illumination in Tris-acetate-phosphate medium at 25 °C with constant mixing (170 rpm) on an incubator shaker (Minitron, INFORS HT). The measurements were performed on the cells in a mid-logarithmic phase of growth, concentrated to OD_800nm_ ≤ 2. *C. reinhardtii* cells were first pelleted (3 min, 4000 rpm) and then resuspended in a fresh TAP medium previously either aerated or bubbled with nitrogen for 1 h. The cells were kept in the dark and further, respectively, either aerated (St1 conditions) or bubbled with nitrogen (St2 conditions) for 45 min (Wollman and Lemaire 1988; Bulté et al. 1990). After this time, a Pasteur pipette was immersed in a sample and immediately quick-frozen in liquid nitrogen. For measurements, the pipette containing sample was quickly placed in a cold finger filled with liquid nitrogen.

### Measurements

**Fig. 1:**
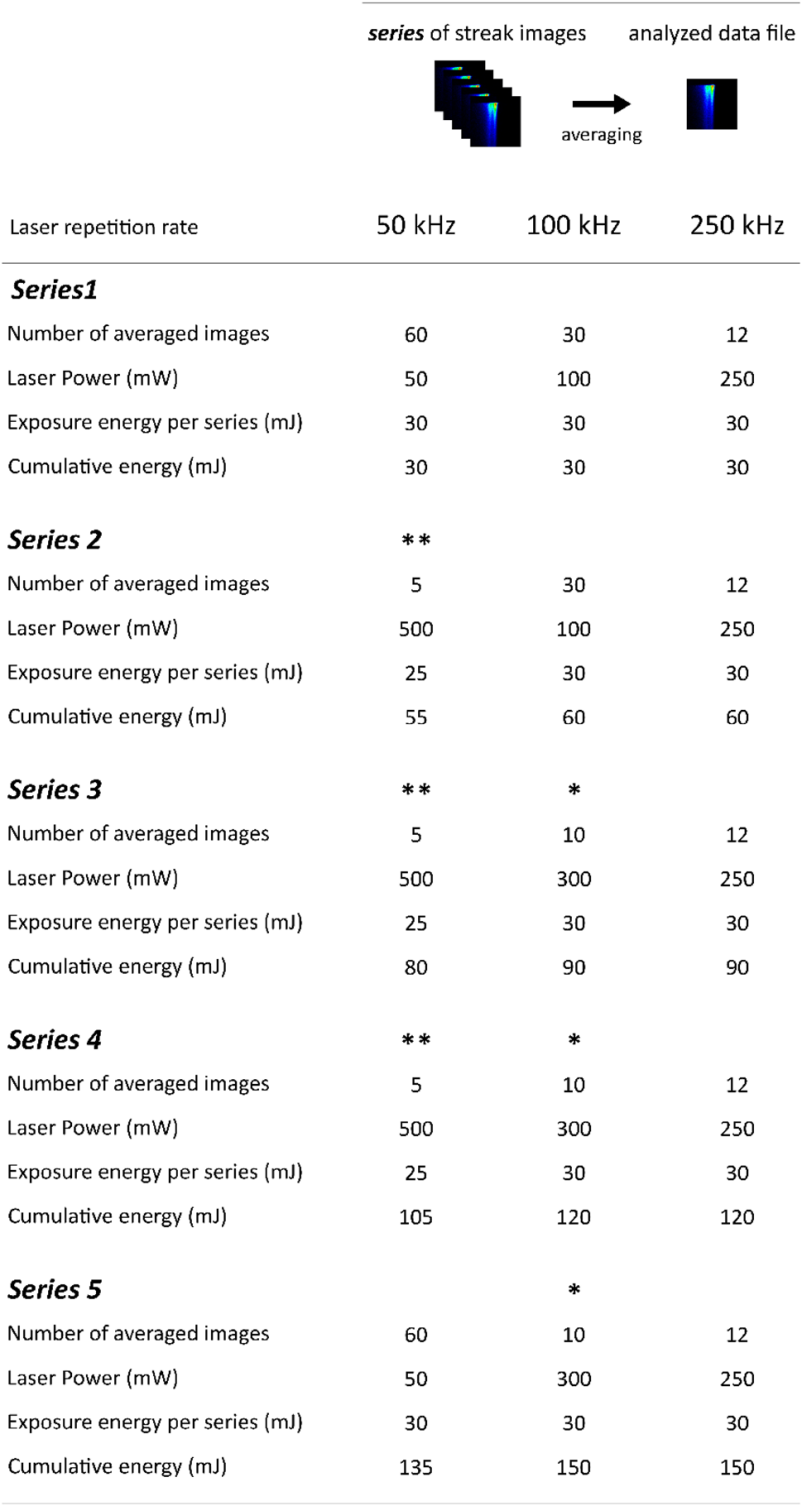
Scheme of data preparation for global and target analysis. Detailed description in the Materials and methods

The full-spectrum time-resolved fluorescence emission was measured in *C. reinhardtii* cells at 77 K by means of the streak camera setup described in detail elsewhere (Gobets et al. 2001). The excitation laser pulses (≈ 100 fs) at 400 nm were generated using a tandem consisting of Vitesse Duo (Coherent, Santa Clara, California), regenerative amplifier RegA 9000 (Coherent, Santa Clara, California), and optical parametric amplifier (OPA, Coherent, Santa Clara, California). The diameter of the laser beam on the sample was 1 × 1.3 mm. In order to check the influence of singlet–triplet formation on the quenching effect, three laser repetition rates were used: 50, 100 and 250 kHz. The energy per pulse was either 1 nJ, or 3 nJ (series indicated with * in Fig. 1), or 10 nJ (series indicated with ** in Fig. 1). The resulting fluorescence light was first focused on the input slit (100 µm) of a spectrograph (Chromex 250IS, 50 grooves/mm ruling, blaze wavelength 600 nm, spectral resolution of 2 nm), subsequently on the input slit (40 µm) and finally on the photo-cathode of the streak camera Hamamatsu C5680 mounted with the M5675 Synchroscan unit and the Digital CCD Camera Hamamatsu Orca R2. For spectral calibration, an Argon lamp (Oriel Instruments Argon lamp model 6030) was used, while spectro-temporal correction was performed by means of a xenon lamp providing homogeneous white light (Osram, HLX 64642 150W 24 V). The spectral range of measured fluorescence spanned from 550 to 820 nm with a wavelength resolution of ≈ 4 nm. The time window for acquisition was ≈ 1.5 ns with a temporal resolution of ≈ 20 ps.

Each streak image was acquired for 10 s (Fig. 1). The images were next corrected for background, the wavelength-dependent sensitivity of the detector (shading) and jitter, using the Hamamatsu High Performance Digital Temporal Analyzer (HPD-TA) 8.2.0. The series of images were averaged according to scheme depicted in Fig. 1, to yield data files which corresponded to a condition when a sample was exposed to an energy of 30 or 25 mJ. This energy is named hereafter *exposure energy per series*. Note that, an energy per series equal to 25 mJ is indicated with ** in Fig. 1. The total energy to which a sample had been exposed since the beginning of illumination is named hereafter *cumulative exposure energy*. We estimate the number of excitations per second per PSII supercomplex to be around 110 at 50 kHz, 220 at 100 kHz and 550 at 250 kHz, the detailed calculation is presented in the Supplementary Information (section “Quencher vs. singlet–singlet annihilation”).

Because in the time of the measurements the quencher was being created, each analysed data file describes an average effect of quencher accumulation on the fluorescence dynamics.

### Analysis

The time-resolved fluorescence measured in the intact *C. reinhardtii* cells was first analysed globally (Van Stokkum et al. 2004) using the Glotaran software (Snellenburg et al. 2012) (version 1.3). Briefly, each fluorescence intensity decay $\psi \left( {t,~\lambda } \right)$ was simultaneously fit at all measured wavelengths (λ) with a sum of four exponentially decaying components, characterized by rate constant ${k_i}=\tau _{i}^{{ - 1}}$, which were convolved with the instrument response function (IRF):

$$\psi \left( {t,~\lambda } \right)=\mathop \sum \limits_{{i=1}}^{4} [\exp \left( { - {k_i}t) \otimes {\text{IRF}}\left( t \right)} \right]{\text{DA}}{{\text{S}}_i}(\lambda ).$$

The decay-associated spectra (DAS) describe how the amplitude of each fitted component changes as a function of the detection wavelength. Datasets obtained at a specific laser repetition rate were simultaneously analysed globally (Fig. 1), and the kinetic components were linked whereas the DAS were independent between the datasets.

Steady-state fluorescence spectra were reconstructed from the time-resolved fluorescence according to the following equation: $\psi \left( \lambda \right)=\Sigma {\text{DA}}{{\text{S}}_i}(\lambda ){\tau _i}$. The resulting spectra were normalized using a scaling factor obtained from the total integrated area under the IRF calculated upon the full-spectrum global analysis of the time-resolved fluorescence data.

In order to describe the excitation energy transfer between different functional states in the pigment-protein complexes and the decay of each of these compartments, target analysis was applied (Holzwarth 1996; Van Stokkum et al. 2004). We used the functional compartmental model of excitation energy kinetics in *Chlamydomonas reinhardtii* wild-type and photosynthetic mutants at 77 K (Wlodarczyk et al. 2016), proposed in our previous work (Snellenburg et al. 2017). The Species-Associated Spectra (SAS) resolved therein served as a priori information in the fitting of the current data. For the details regarding the construction of the functional compartmental model including imposed spectral constraints, we refer the reader to (Snellenburg et al. 2013). With an exception of the quenching rate constant *Q*, all other rate constants were fixed as in Fig. S4 in (Snellenburg et al. 2017).

## Results

### 77 K steady-state fluorescence emission

**Fig. 2:**
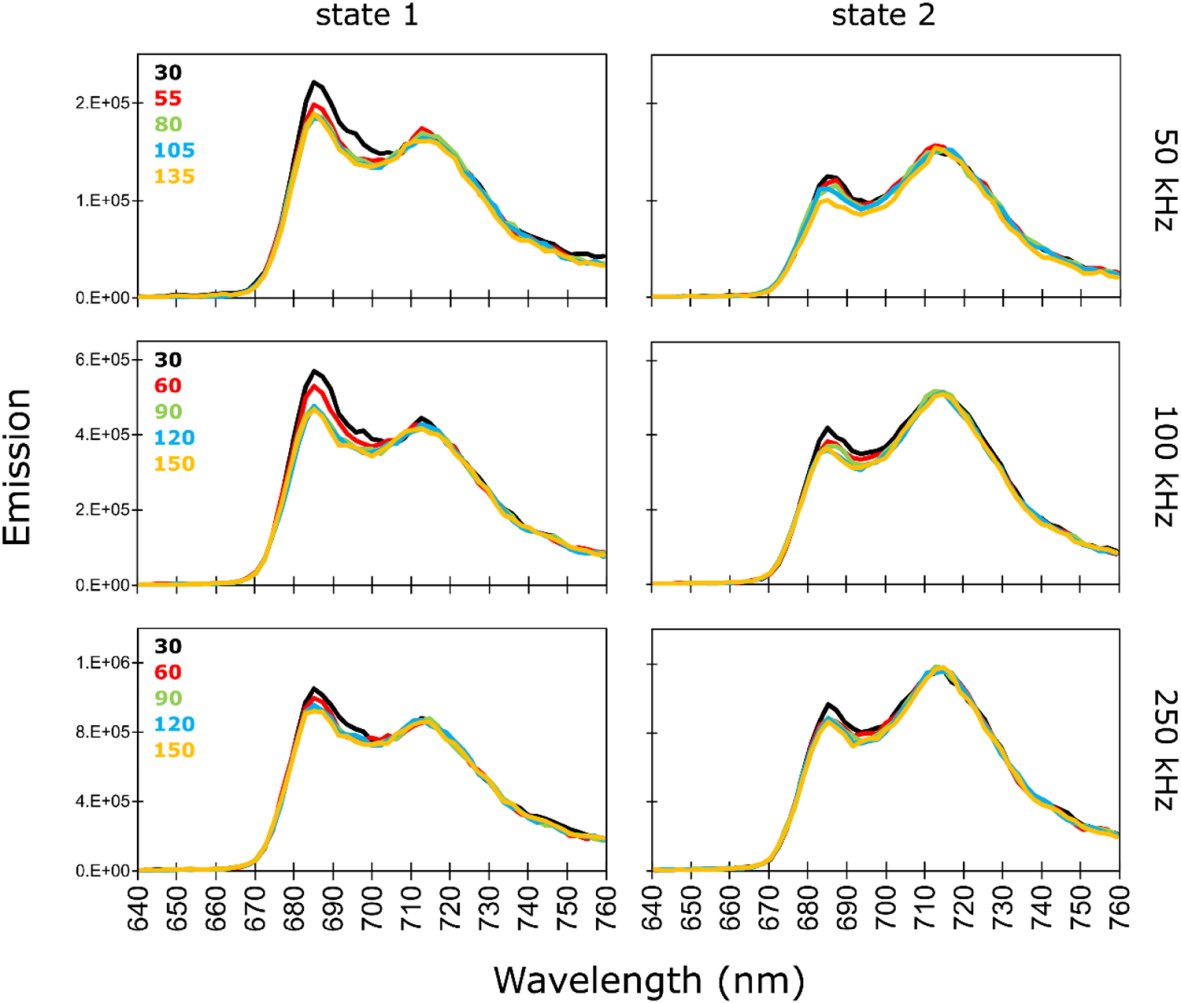
77 K steady-state fluorescence spectra reconstructed from globally analysed time-resolved fluorescence measured upon excitation at 400 nm in *C. reinhardtii* WT after incubation for 45 min under St1 conditions (left panel) or under St2 conditions (right panel). The spectra were normalized using a scaling factor obtained upon global analysis (more details in Materials and Methods). For each laser repetition rate, state 1 and state 2 spectra have the same vertical axis, and different colours of the spectra represent different cumulative exposure energies indicated in mJ (Fig. 1)

Exposure of the *C. reinhardtii* cells frozen to 77 K to prolonged excitation light decreases their fluorescence emission between 683 and 704 nm which primarily originates from PSII (Fig. 2). On the contrary, the PSI emission peak around 715 nm is barely affected (note that spectra in Fig. 2 were not normalized at this wavelength). These observations hold for all three excitation laser repetition rates used in this study. The extent of the observed changes can be more accurately estimated from the areas under the emission spectra ranging from 640 to 760 nm. At the intermediate excitation laser repetition rate (100 kHz), the decrease of this parameter upon illumination is 8% in St1 versus 5% in St2. The respective declines are somewhat stronger at 50 kHz, with 10% in St1 versus 7% in St2, while the smallest change is observed at 250 kHz with a decline of 4% in both states.

### 77 K time-resolved fluorescence: global analysis

**Fig. 3:**
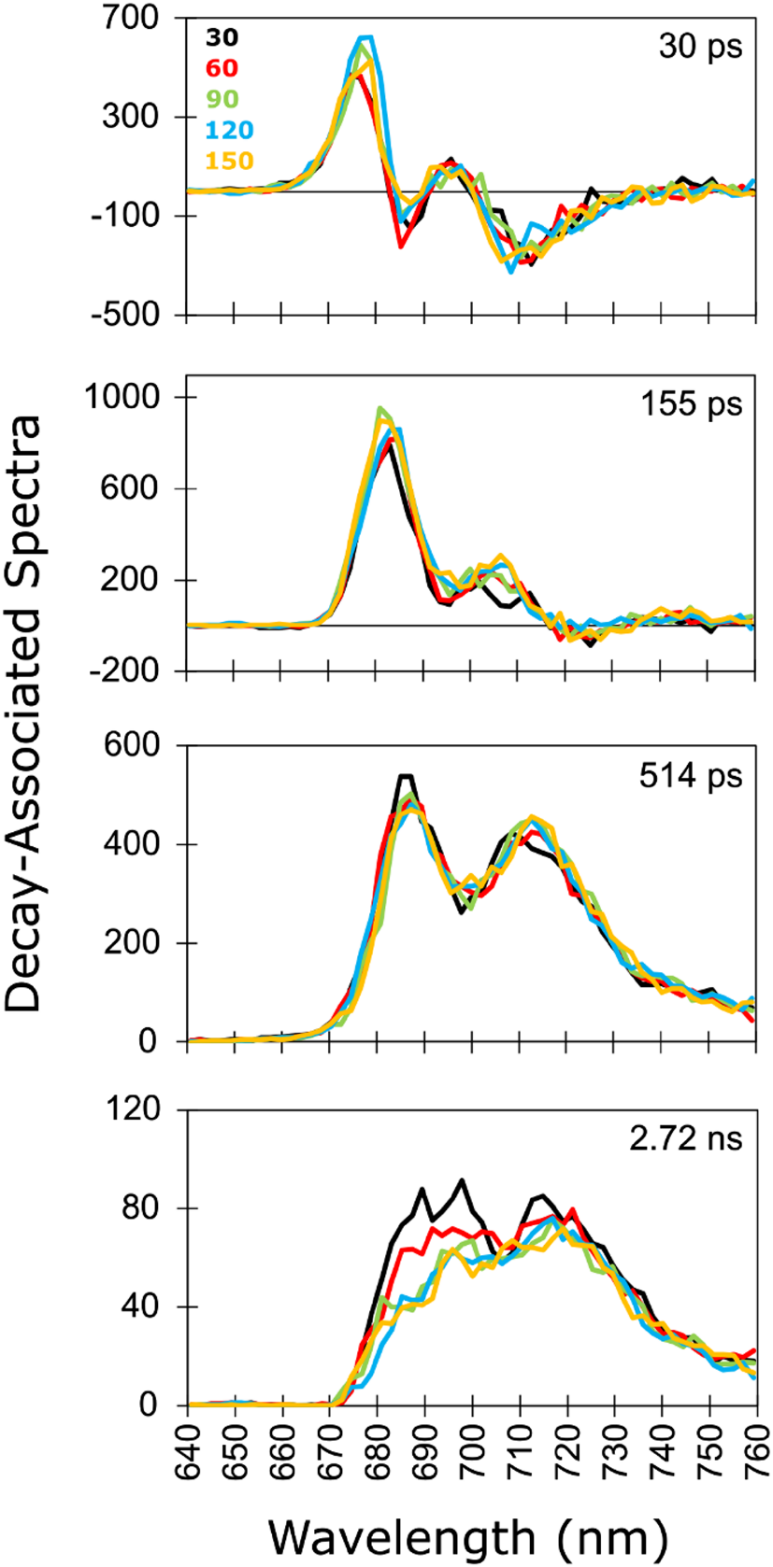
Decay-associated spectra (DAS) resulting from global analysis of 77 K time-resolved fluorescence measured upon excitation at 400 nm with laser repetition rate of 100 kHz in *C. reinhardtii* WT cells after incubation under St1 conditions. Different colours of the spectra represent different cumulative exposure energies indicated in mJ (Fig. 1)

Globally analysed 77 K time-resolved fluorescence of *C. reinhardtii* WT was adequately fitted with four components (Fig. 3, Fig. S1A, B, C). The number of components and their interpretation is the same as in our previous work (Wlodarczyk et al. 2016), where the laser repetition rate was 250 kHz and the cumulative energy was 150 mJ. For comparison, in the current study, 150 mJ (or 135 mJ at 50 kHz) was accumulated upon measurement of the final series (Series 5 in Fig. 1) and it is indicated in yellow in Fig. 3. DAS for measurements in St2 cells at 100 kHz (Fig. S1A) as well as in St1 and St2 cells at 50 kHz (Fig. S1B) and at 250 kHz (Fig. S1C) laser repetition rates are depicted in the Supplementary Information.

Series 1–5 (Fig. 1) measured at a specific laser repetition rate were analysed with linked lifetimes and independent decay-associated spectra (DAS). At 100 and 50 kHz laser repetition rates the largest effect of prolonged illumination of frozen *C. reinhardtii* WT cells occurs in the amplitude of the St1 DAS characterized by the lifetime of 2.7 ns and the peak around 690 nm (Fig. 3, Fig. S1A, B). Trapping on PSII on this time scale [the major contributor to the F685 and F695 emission bands (Andrizhiyevskaya et al. 2005)] clearly changes under prolonged exposure to measuring light. This component is therefore responsible for most of the changes in the steady-state fluorescence spectra shown in Fig. 2. This observation is in line with the previous global analysis of light-induced low-temperature quenching in isolated PSII-enriched membranes of spinach (Mohamed et al. 2016) and isolated PSII complexes of spinach and cyanobacterium *Synechocystis* (Schweitzer et al. 1998).

At the highest repetition rate of 250 kHz, the effect of prolonged illumination on the DAS is significantly smaller than at 50 or 100 kHz (Fig. S1C). We observe moreover no significant effect of prolonged illumination on the respective lifetimes at the different laser repetition rates.

Comparison of state 1 versus state 2 cells indicates that the effect of prolonged illumination is somewhat stronger in the former (Fig. S1A, B, C).

In global analysis, the fluorescence decay traces are fitted at each wavelength with a specific number of components decaying exponentially in parallel. It gives therefore only a rough overview of the excitation energy kinetics in the photosynthetic apparatus. In order to resolve the sequence of excitation energy transfer events and to estimate equilibria between the different compartments we apply target analysis.

### 77 K time-resolved fluorescence: target analysis

**Fig. 4:**
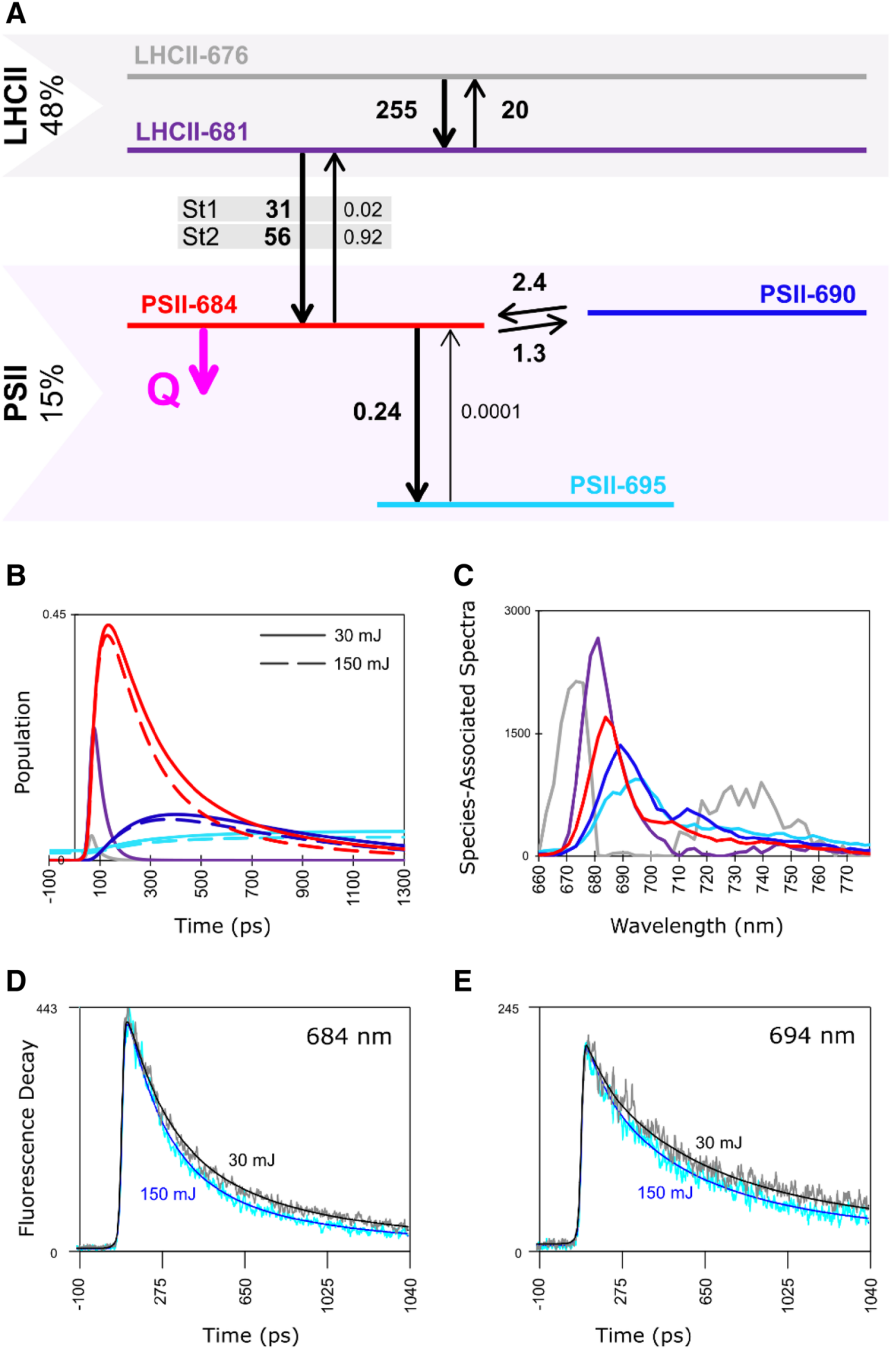
Target analysis of 77 K time-resolved fluorescence measured in *C. reinhardtii* WT cells in St1. The cells were continuously illuminated with 400 nm laser light at 100 kHz repetition rate. **a** Compartmental model for the LHCII–PSII complex. The population directly after excitation per subunit PSII (15%) and LHCII (48%) is indicated (Table S2). The colour key of the compartments in **a** is used in **b** and **c**. Numbers next to the black arrows indicate rate constants in ns^− 1^. The natural decay rate constant is 0.2 ns^− 1^ for all compartments (omitted for clarity). The light-induced quenching rate constant Q (indicated in magenta) increases from 3.3 to 4.2 ns^− 1^ with cumulative exposure energy 30 to 150 mJ. **b** Population dynamics in the LHCII–PSII complex with cumulative exposure energy 30 mJ (solid) or 150 mJ (dashed). **c** Estimated SAS of each compartment. Emission of PSII-690 and PSII-695 above 700 nm is discussed in the Supplementary Information. **d** Time-resolved emission at 684 nm and **e** at 694 nm. Traces are scaled to their maximum. Key: accumulated energy 30 mJ (data—grey; fit—black, Q = 3.3 ns^− 1^), 150 mJ (data—cyan; fit—blue, Q = 4.2 ns^− 1^)

The functional compartmental model applied to characterize the quenching observed in the current study has been developed before to describe the excited state dynamics of LHCII-LHCI-PSI and LHCII–PSII complexes in *Chlamydomonas reinhardtii* in situ at 77 K (Snellenburg et al. 2017). The compartmental model for the LHCII–PSII complex is depicted in Fig. 4. The compartmental model for the LHCII–LHCI–PSI complex, the resulting population profiles and SAS are depicted in Fig. S2. The guidance SAS, taken from our previous work (Snellenburg et al. 2017) and the SAS estimated from the current modelling are very similar (Fig. S3).

The PSII complex in the model is described by five compartments: two compartments of light-harvesting complex II: LHCII-676, LHCII-681, and three compartments of the PSII core: PSII-684, PSII-690 and PSII-695 (Fig. 4). At laser repetition rates of 50, 100 and 250 kHz the resulting population profiles (Fig. 4b, Fig. S4A, C) obtained for the cumulative energy of 150 mJ (or 135 mJ at 50 kHz) as well as species-associated spectra (Fig. 4c, Fig. S4B, D) are similar to our previous results which were obtained at 250 kHz laser repetition rate and with a cumulative energy of 150 mJ (Snellenburg et al. 2017). The consequence of the fact that the data files analysed previously were obtained upon averaging of all measured streak images (no division in series) will be discussed below.

**Fig. 5:**
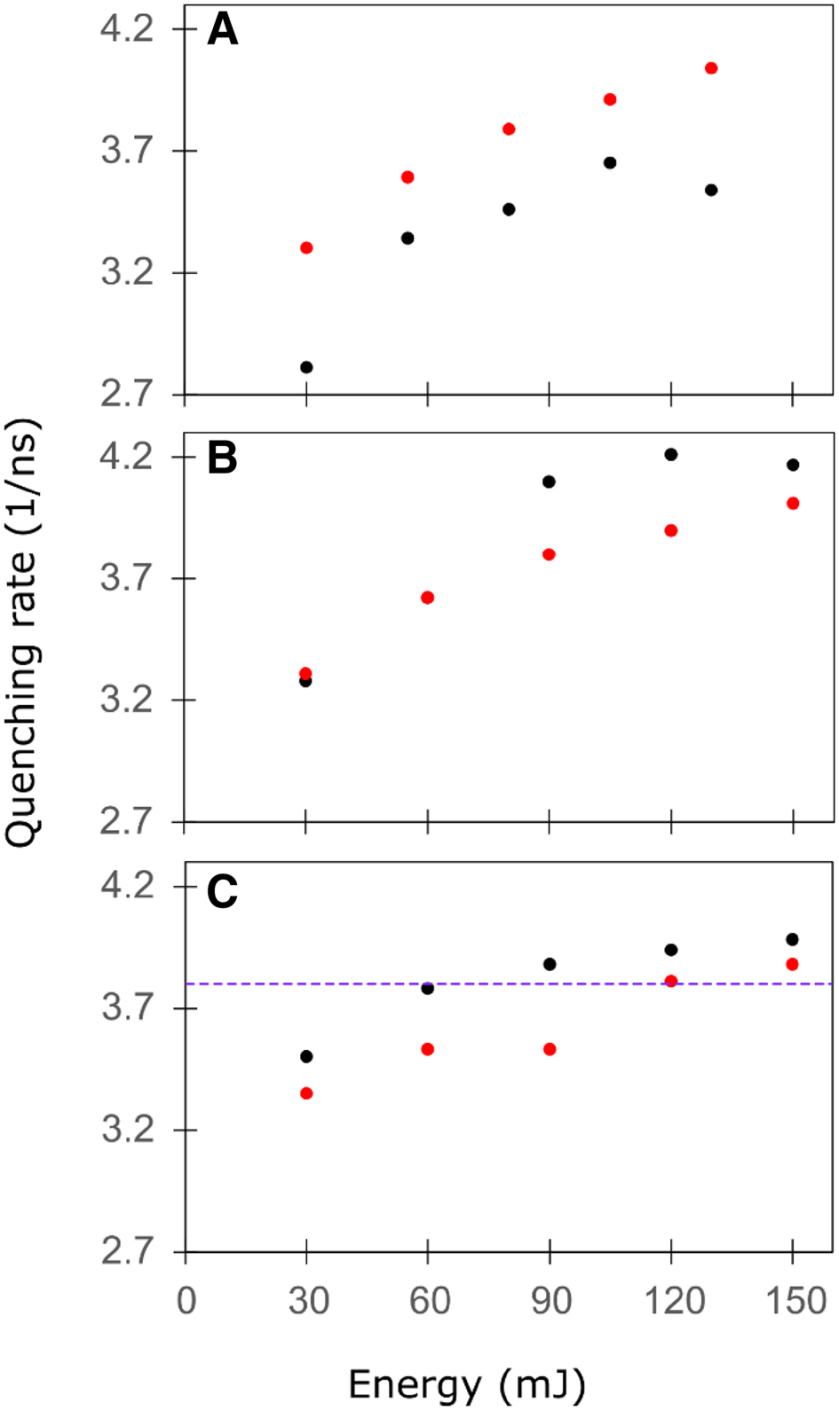
Quenching rate constant Q as a function of the cumulative exposure energy upon prolonged illumination of *C. reinhardtii* WT cells in St1 (black) or in St2 (red). The laser repetition rate was 50 kHz (**a**), 100 kHz (**b**) or 250 kHz (**c**). Values of Q and error estimates are listed in Table S1. The quenching rate constant of 3.8 ns^− 1^ estimated in our previous study (Snellenburg et al. 2017) is indicated with a violet dashed line in C

Upon prolonged illumination of the *C. reinhardtii* cells frozen to 77 K, we observe changes in the population profiles of the PSII compartments, most prominently in the PSII-684 compartment (red curves in Fig. 4b, Fig. S4A, C). Shortening of the fluorescence lifetimes is moreover visible in the time-resolved emission traces in the PSII-dominated emission range both in St1 and in St2 at all laser repetition rates (Fig. 4d, e, Fig. S5A, B, C). This effect is caused by the quenching rate constant Q on the PSII-684 compartment, increasing upon prolonged illumination at all three laser repetition rates used in this work (Fig. 4, Table S1). This quenching rate constant was resolved in our previous study using a 250 kHz laser repetition rate with a cumulative energy of 150 mJ (Snellenburg et al. 2017); however, there each analysed data file was obtained upon averaging of all streak images acquired in one experiment. Here, in order to observe the quencher accumulation, we analysed five data files where each data file (referred here to as “series”) resulted from averaging of a specific number of subsequent streak images (Fig. 1). As expected, the quenching rate constant obtained in the previous work (3.8 ns^− 1^) is close to an average value of the quenching rate constants resolved currently upon quencher accumulation (Fig. 5c).

The quenching rate constant Q resolved from the first measurement in each series of five has the lowest value in St1 cells measured at 50 kHz (2.8 ns^− 1^) and in all other cases it is around 3.3 ns^− 1^. As the illumination continues, Q increases and reaches maximally 4.2 ns^− 1^ for St1 cells measured at 100 kHz and somewhat less in other measurements. The largest increase in Q is observed also in St1 cells at 100 kHz and equals 0.93 ns^− 1^.

The amplitude matrices from the target analysis (estimated from the 77 K time-resolved fluorescence measured with a laser repetition rate of 100 kHz) of the LHCII–PSII complexes in St1 are given in Table 1 for series 1 and Table 2 for series 5, respectively. They are interpreted as follows: first LHCII-676 and LHCII-681 equilibrate in 3.6 ps, and in 35 ps the LHCII equilibrates with PSII. Upon quencher accumulation, the dominant decay lifetime of PSII decreases from 173 to 154 ps, and the next important decay lifetime of PSII decreases from 615 to 555 ps. Finally, the amplitude of the 4998 ps lifetime that represents PSII-695 decreases from 0.046 to 0.036, expressing that the amount of long-lived fluorescence decreases when Q increases. Altogether these changes excellently describe the observed changes visible in Fig. 4d, e and Fig. S5A.

**Table 1:** Amplitude matrix of the target analysis of the LHCII–PSII complexes in situ at 77 K

Lifetime (ps)	3.6	35	173	615	4998
LHCII-676	0.22	0.04	0	0	0
LHCII-681	− 0.24	0.46	0	0	0
PSII-684	0.03	− 0.61	0.57	0.15	0
PSII-690	0	0.03	− 0.23	0.20	0
PSII-695	0	0.005	− 0.025	− 0.026	0.046

Kinetic scheme of the species and estimated microscopic rates are given in Fig. 4. Series 1 for St1 cells measured at 100 kHz laser repetition rate, quenching rate constant Q = 3.3 ns^− 1^. Further explanation in the text

**Table 2:** Amplitude matrix of the target analysis of the LHCII–PSII complexes in situ at 77 K

Lifetime (ps)	3.6	35	154	555	4998
LHCII-676	0.22	0.04	0	0	0
LHCII-681	− 0.24	0.46	0	0	0
PSII-684	0.03	− 0.63	0.64	0.11	0
PSII-690	0	0.03	− 0.21	0.18	0
PSII-695	0	0.005	− 0.025	− 0.017	0.036

Kinetic scheme of the species and estimated microscopic rates are given in Fig. 4. Series 5 for St1 cells measured at 100 kHz laser repetition rate, quenching rate constant Q = 4.2 ns^− 1^. Further explanation in the text

As indicated in the global analysis (Fig. 3), no light-induced quenching is observed on the PSI compartments resolved upon the target analysis. The obtained PSI population dynamics (Fig. S2B) and the PSI SAS (Fig. S2C) are in line with previous modelling results (Snellenburg et al. 2017). The state transitions behaviour is also reproduced in the current measurements and well fitted with the model (Fig. S5A, B, C, Table S2).

## Discussion

Light-induced quenching at cryogenic temperatures was observed before in various preparations containing PSII (Kyle et al. 1983; Schweitzer and Brudvig 1997; Schweitzer et al. 1998; Siffel et al. 2000; Litvín et al. 2005; Mohamed et al. 2016). Kyle et al. (1983) noted that 77 K steady-state fluorescence of PSII, and to lesser extend of PSI, decreases upon prolonged illumination. Global analysis of time-resolved fluorescence measured at 85 K by Schweitzer and Brudvig (1997) revealed that prolonged illumination of frozen PSII lowers the yield of the two ns-components centred around 690 nm. Decreased yield of one ns-component peaking around 692 nm was moreover indicated in a time-resolved study by Mohamed et al. (2016). Our results present in the current work are in line with these previous findings: upon prolonged illumination of *C. reinhardtii* cells frozen to 77 K, we observe that the yield of the PSII-dominated emission band in the component characterized by a lifetime of 2.7 ns drops (Fig. 2). This observation holds independently of the laser repetition rate used to excite a sample, though the effect at 250 kHz is smaller than at 50 and 100 kHz (Fig. S1 A, B and C and Fig. S5 A, B and C). The effect is clearly manifested in steady-state spectra (Fig. 2) and in population profiles resulting from our target analysis (Fig. 4b, Fig. S4 A and C). Though the quencher in our model is localized on the PSII-684 compartment, PSII-690 and PSII-695 are also affected by quencher accumulation as they are functionally connected to PSII-684.

In a recent study on low-temperature light-induced quenching in PSII-enriched membranes, Mohamed et al. (2016) proposed a target model in which the fluorescence decay of the red chlorophyll on CP47 is shortened by a quencher (assumed as Chl_Z_^+^), while the bulk chlorophylls in CP43 and RC are not affected by the quencher. When we include additional decay rate constants on the red chlorophyll in our model (PSII-690 and PSII-695) with a starting value of 0.2 ns^− 1^, instead of increasing upon prolonged illumination, the rate constants decreased, and thus our data do not show any evidence of quenching of the red chlorophyll on CP47. A possible reason why Mohamed et al. observed such a quenching channel is the trapping rate constant of 22 ns^− 1^ on their RP1 compartment. In this case, the only other chlorophyll in the core that can be quenched is the red chlorophyll. In our opinion, this extremely large trapping rate constant is unrealistic at 77 K. Additionally, we have investigated an alternative model derived from Fig. 4a, i.e., that the quencher does not reside at PSII-684, but instead resides at the red Chl compartments PSII-690 and PSII-695 (call this decay rate constant Qred). For all data, we tested this alternative model. Again a dose dependence of Qred appears, cf. Fig. S7, indicating a systematic increase of Qred with prolonged illumination. We established that with an average rate constant of PSII-684, Qred must drop below 0.2/ns at low energies, thus causing unacceptable residuals, and an increase in the relative rms error of the fit by more than 1%. For these reasons, we reject this alternative model.

Measurements at different laser repetition rates revealed that, at least up to 150 mJ energy received by a sample, the light-induced low-temperature quenching is the weakest at the highest repetition rate of 250 kHz (Figs. 2, 5). We propose that this effect is caused by a quenching mechanism that slows down the accumulation of a quencher in the PSII core. It has been shown that upon illumination a triplet state can be created on a carotenoid and its lifetime is < 10 µs (Peterman et al. 1995; Gruber et al. 2015; Santabarbara et al. 2015). Moreover, formation of chlorophyll triplet states has been observed at cryogenic temperatures. Their reported lifetimes depend on temperature and redox conditions and vary between a few µs (van Mieghem et al. 1995; Feikema et al. 2005), 20 µs (van Mieghem et al. 1995), 50–150 µs (Santabarbara et al. 2002) up to 1–2 ms (Santabarbara et al. 2002, 2003, 2007). Taking into account these triplet states lifetimes, we expect significant singlet–triplet annihilation at 250 kHz laser repetition rate, less at 100 kHz and the least at 50 kHz. Even the longest lifetimes of the triplet states (ms) are many orders of magnitude shorter than the acquisition time of the data (minutes), therefore singlet–triplet annihilation will not change the fluorescence decay in the considered times of quencher accumulation. It is possible that further illumination would result in even higher and similar quenching rate constants Q at all laser repetition rates (Schweitzer and Brudvig 1997). As described in the Supplementary Information, singlet–singlet annihilation is negligible in the present work.

Kyle et al. (1983) observed that quenching induced upon illumination in spinach leaves at 77 K is more pronounced in St1 than in St2. Two major reasons would lead to this result. Firstly, upon the same illumination, a quencher will accumulate faster when the antenna of PSII is larger, i.e., in St1. The second reason is related to the observation that excitation from one supercomplex can be trapped on another one (Haferkamp et al. 2010; Stirbet 2013), and thus once the quencher is created, its effective quenching range will be larger in St1 where more PSII complexes are interconnected creating megacomplexes, in opposition to St2 in which the PSII complexes are connected more weakly (Iwai et al. 2008). In the current work, we observe that the quenching rate constant Q increases strongly in the initial phase of quencher accumulation. As the illumination continues the increase of Q lessens, possibly reaching saturation (Fig. 5). This trend is similar in St1 and in St2; and however, it is not obvious that Q is larger in St1 than in St2. Schweitzer et al. observed that the quencher primarily affects chlorophylls in the core antenna and less in the peripheral antenna, which might explain why we do not observe a very strong effect of the state transition on the quenching rate constant Q.

In our earlier studies on state transitions, we used a 250 kHz laser repetition rate and the maximum of cumulative energy was 150 mJ (Wlodarczyk et al. 2016; Snellenburg et al. 2017). While in the current work we averaged a series of streak images at different times of experiment to get the information about the quencher accumulation process, the previous data were a result of averaging of all streak images accumulated during the complete experiment. As expected, the quenching rate constant obtained in the previous study is close to an average quenching rate constant in the current work, namely 3.8 ns^− 1^ (Fig. 5). We now show that also with a lower cumulative energy, the state transitions behaviour is reproduced and our previous interpretation of the results remains valid. In the Supplementary Information, we discuss differences in low-temperature steady-state spectra of *C. reinhardtii* cells measured upon different ST induction conditions.

Quenching induced in the present experiments was not changing when the sample was incubated in darkness for 30 min (Fig. S6). It means that the quenching effect does not diminish when the sample is not illuminated, as was also shown in the previous work of Kyle et al. (1983).

It has been observed that photoinhibition (irreversible formation of fluorescence quenching) due to illumination decreases at lower temperatures (Schweitzer and Brudvig 1997). Moreover, inhibition of O_2_ evolution correlated with photoinhibition was very low below 100 K. In another study rewarming of a quencher-containing sample and its renewed freezing to 77 K showed that only a small portion of quenching remained and it was not present in the absorption spectra suggesting that there was no photodestruction of a pigment bed (Kyle et al. 1983). In our experiments, we were not able to assess the reversibility of the quenching, but based on the previous findings we anticipate that it has a minor effect on our conclusions.

## Conclusions and outlook

In this work, we showed accumulation in time of the light-induced 77 K quenching in the intact cells of *C. reinhardtii*. Using the recently developed kinetic model for photosynthetic excitation energy transfer in *C. reinhardtii*, we identified that the quenching occurs on bulk chlorophyll(s) residing in the PSII-684 compartment and it affects also emission from the red pigment on PSII (PSII-690 and PSII-695). We conclude that at least in part of our earlier data on state transitions, the presence of quenching was highly possible, nevertheless state transitions estimates obtained from that data remain valid.

Initially, our experiment was designed to investigate an interesting idea of Schweitzer and Brudvig (1997): “the presence of a well-defined quencher in PSII may make it possible to study the connectivity between antenna systems in different sample preparations.” Our measurements on the intact cells of *C. reinhardtii* described in this work did not provide a clear proof that different antenna sizes related to state transitions influence the quencher dynamics. We propose that the dependence is so subtle that besides a well-defined quencher it requires also a well-defined (uniform) and simpler sample, for example isolated PSII complexes with different antenna sizes.

## Electronic supplementary material

Below is the link to the electronic supplementary material.

Supplementary material 1 (DOCX 3723 KB)
