# Supplementary material for: Development of fluorescence quenching in Chlamydomonas reinhardtii upon prolonged illumination at 77 K
Source: Photosynth Res. 2018 Jun 13;137(3):503–13. doi: 10.1007/s11120-018-0534-8 (PMC6182390; doi:10.1007/s11120-018-0534-8)
Supplement: Supplementary file 1 — Supplementary material 1 (DOCX 3723 KB) [file 11120_2018_534_MOESM1_ESM.docx]

# Supplementary Material

Development of fluorescence quenching in *Chlamydomonas reinhardtii* upon prolonged illumination at 77 K

*Lucyna M. Wlodarczyk, Joris J. Snellenburg, Jan P. Dekker, Ivo H. M. Stokkum*

*LaserLaB, Department of Physics and Astronomy, Faculty of Science,
Vrije Universiteit Amsterdam, De Boelelaan 1081, 1081 HV Amsterdam, The Netherlands*

**
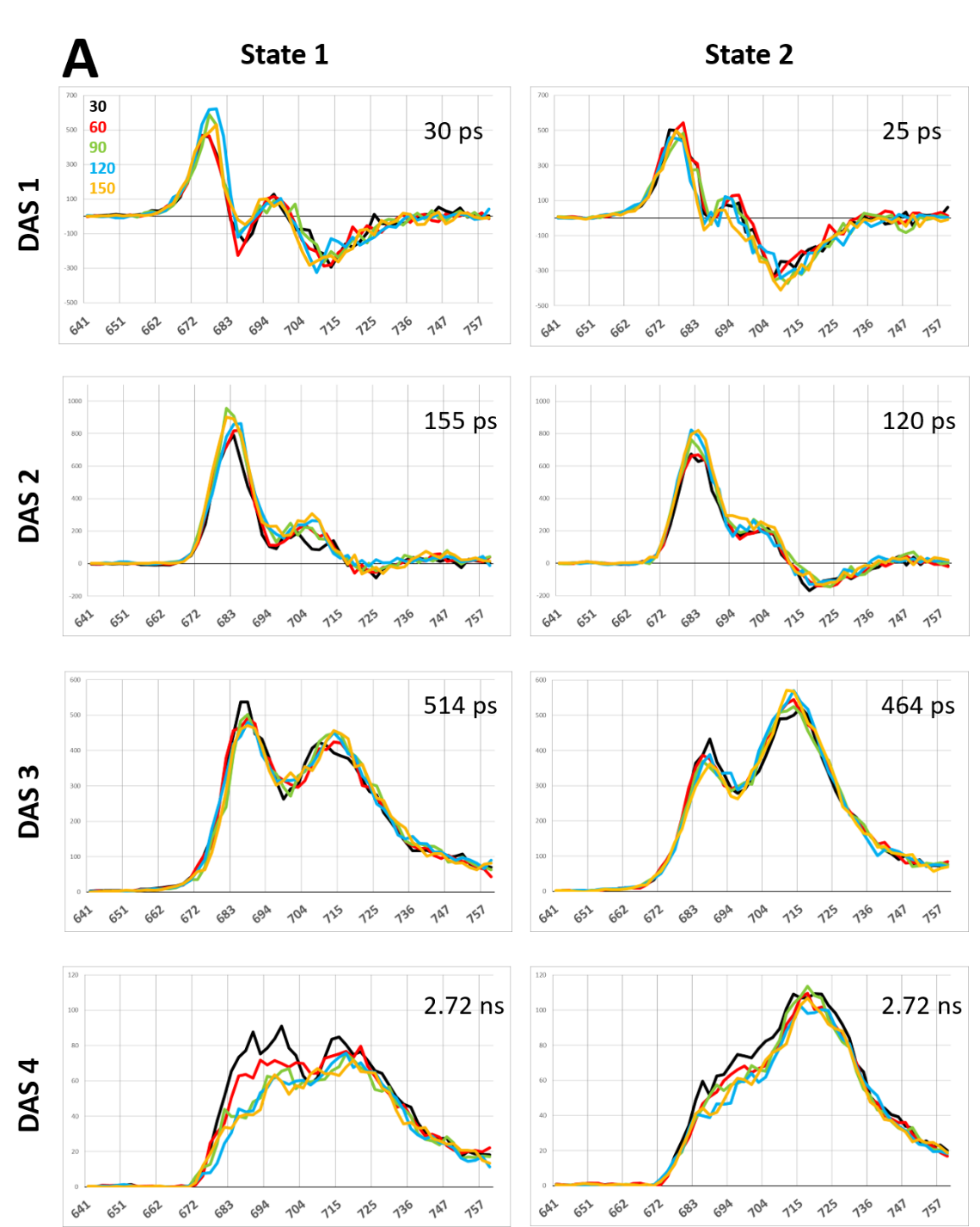
**

**Figure S1A** Decay-Associated Spectra (DAS) resulting from global analysis of 77 K time-resolved fluorescence measured upon excitation at 400 nm with laser repetition rate of 100 kHz in *C. reinhardtii* WT cells after incubation for 45 minutes under St1 conditions (left panels) or under St2 conditions (right panels). Different colours of the spectra represent different cumulative exposure energies indicated in mJ (Materials and Methods, Figure 1).

**
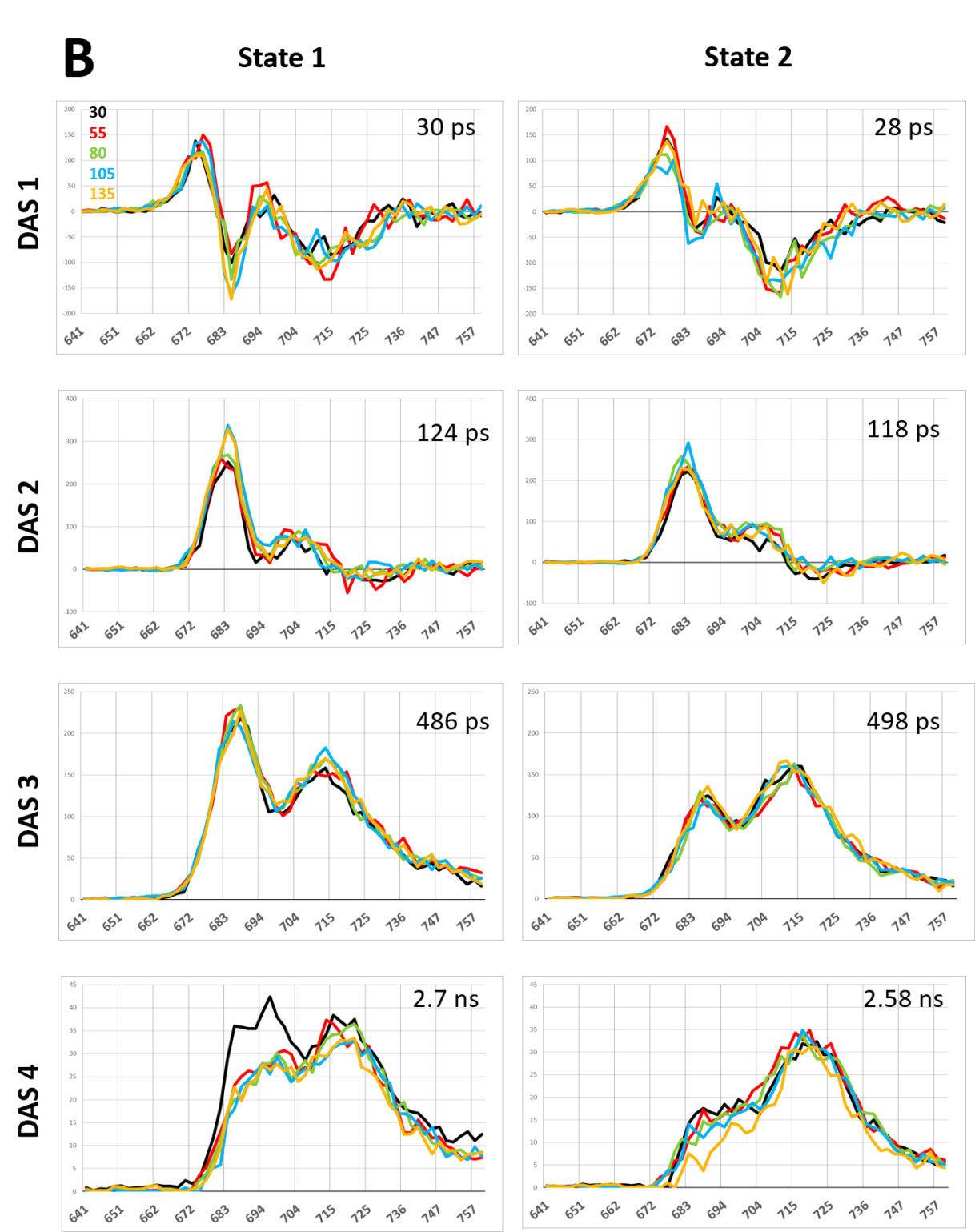
**

**Figure S1B** Decay-Associated Spectra (DAS) resulting from global analysis of 77 K time-resolved fluorescence measured upon excitation at 400 nm with laser repetition rate of 50 kHz in *C. reinhardtii* WT cells after incubation for 45 minutes under St1 conditions (left panels) or under St2 conditions (right panels). Different colours of the spectra represent different cumulative exposure energies indicated in mJ (Materials and Methods, Figure 1).


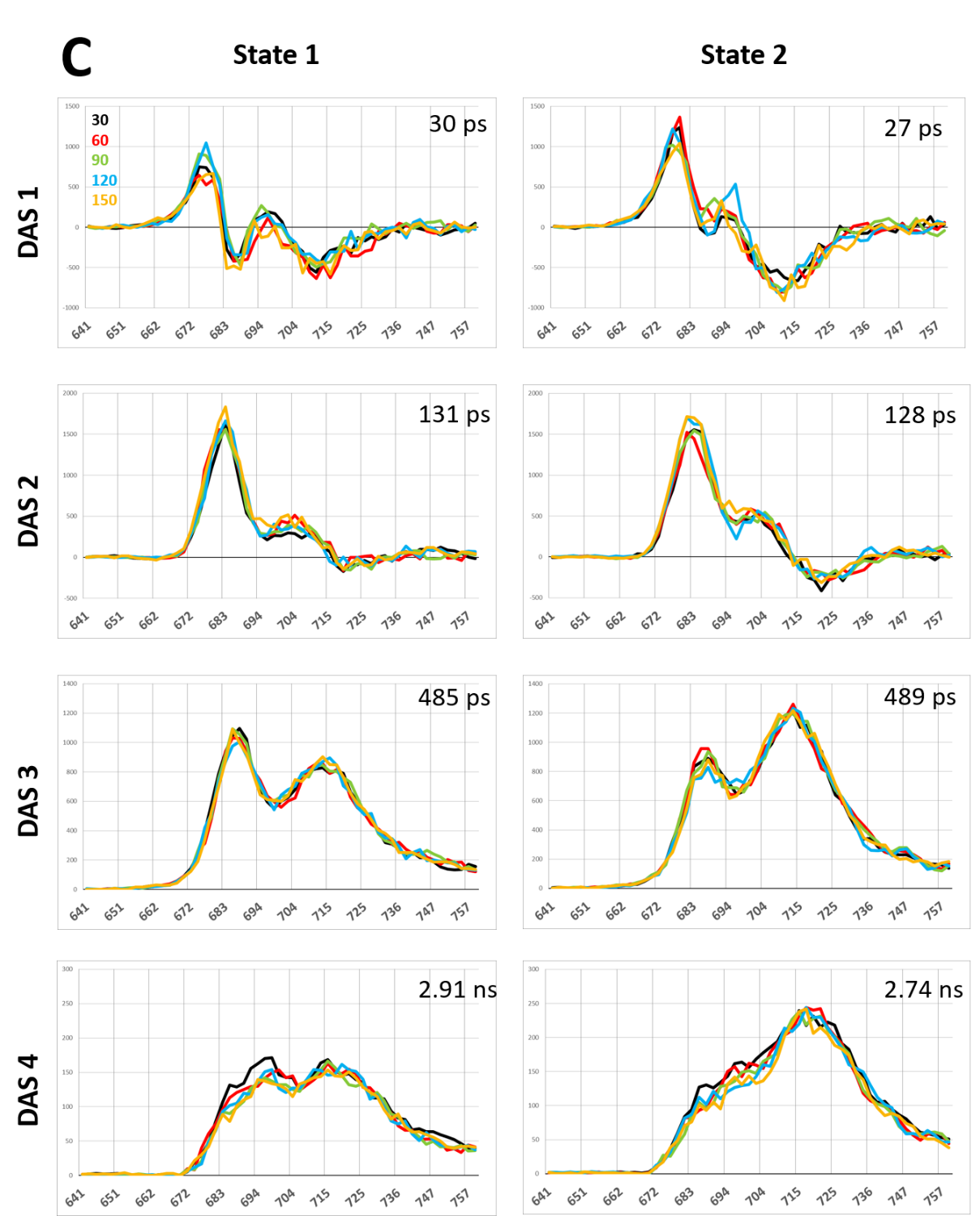


**Figure S1C** Decay-Associated Spectra (DAS) resulting from global analysis of 77 K time-resolved fluorescence measured upon excitation at 400 nm with laser repetition rate of 250 kHz in *C. reinhardtii* WT cells after incubation for 45 minutes under St1 conditions (left panels) or under St2 conditions (right panels). Different colours of the spectra represent different cumulative exposure energies indicated in mJ (Materials and Methods, Figure 1).


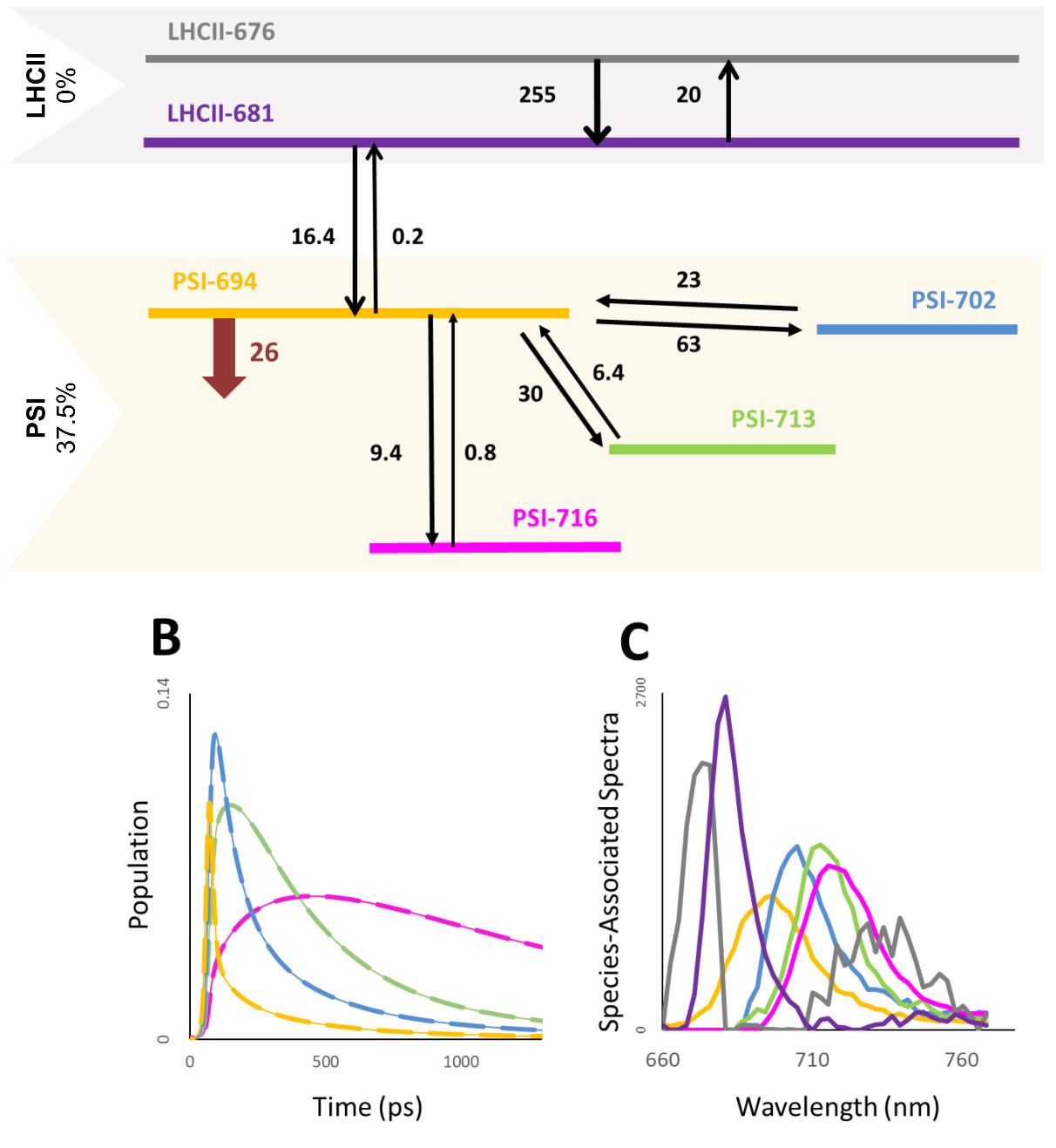


**Figure S2** Results of target analysis of 77 K time-resolved fluorescence measured in *C. reinhardtii* WT cells in St1. The cells were continuously illuminated with 400 nm laser light at 100 kHz repetition rate. A) Compartmental model for the LHCII-LHCI-PSI complex. . The population directly after excitation per subunit PSI (37.5%) and LHCII (0%) in St1 are indicated (Table S2). The colour key of the compartments in A is used in panels B and C. Numbers next to the black arrows indicate rate constants in ns^-1^. The rate constant of photochemical trapping due to charge separation was fixed to 26 ns^-1^ (brown arrow). The natural decay rate constants are 0.3 ns^-1^ for PSI compartments, and 0.2 ns^-1^ for LHCII compartments (omitted for clarity). B) Population dynamics in the LHCII-LHCI-PSI complex in St1 cells upon continuous illumination which resulted in increase of the cumulative energy from 30 mJ (solid) to 150 mJ (dashed). This increase has no influence on the population dynamics in the LHCII-LHCI-PSI complex. C) Estimated SAS of each compartment.

Figure S3 Guide spectra (estimated in (Snellenburg et al. 2017), solid lines) used to estimate the SAS (dashed lines). *C. reinhardtii* WT cells in St1 and in St2. The cells were continuously illuminated with 400 nm laser light at repetition rate of 100 kHz. A) LHCII and PSII SAS, B) normalized residuals from A, i.e. the difference of the estimated SAS and the guide spectra, C) PSI SAS, D) normalized residuals from C. Color key of the species as in Fig.4 and Fig.S2.


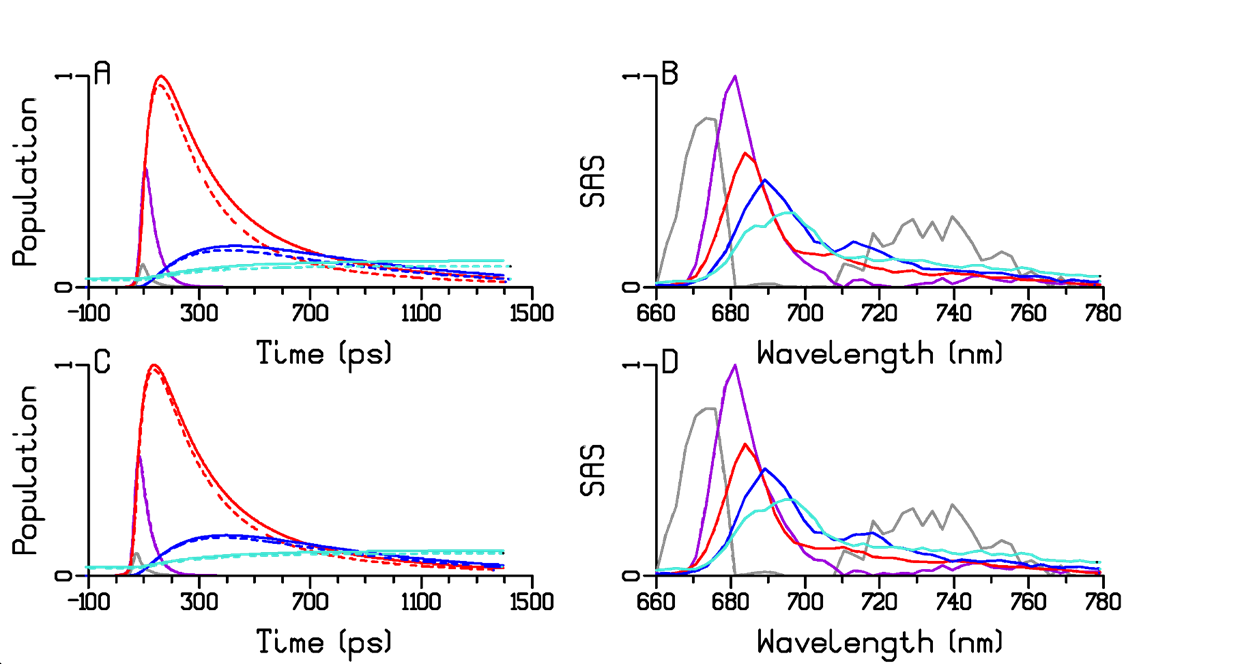


**Figure S4** Results of target analysis of 77 K time-resolved fluorescence measured in *C. reinhardtii* WT cells in St1. The cells were continuously illuminated with 400 nm laser light at repetition rate of 50 kHz (upper panel) or 250 kHz (lower panel). Left panel: population dynamics in the LHCII-PSII complex in St1 cells upon continuous illumination at 50 kHz (A) or 250 kHz (C) which resulted in increase of the cumulative exposure energy in (A) from 30 mJ (solid) to 135 mJ (dashed), and in (C) from 30 mJ (solid) to 150 mJ (dashed). Quenching rate constant Q increases in (A) from 2.8 ns^-1^ to 3.65 ns^-1^ and in (C) from 3.5 ns^-1^ to nearly 4 ns^-1^. Right panel: Estimated SAS of each compartment. The colour key of the population profiles and SAS refers to compartments as in the target model depicted in Figure 4A in the main text.

## Emission of PSII-690 and PSII-695 above 700 nm

Though PSII and LHCII compartments are expected to show emission >700 nm due to vibrational contribution, the relatively high amplitude of the emission of PSII-690 and PSII-695 observed at these wavelength can possibly also be caused by moderately red-shifted chlorophylls (Krausz et al. 2005) that are selectively coupled to PSII-690 and PSII-695.


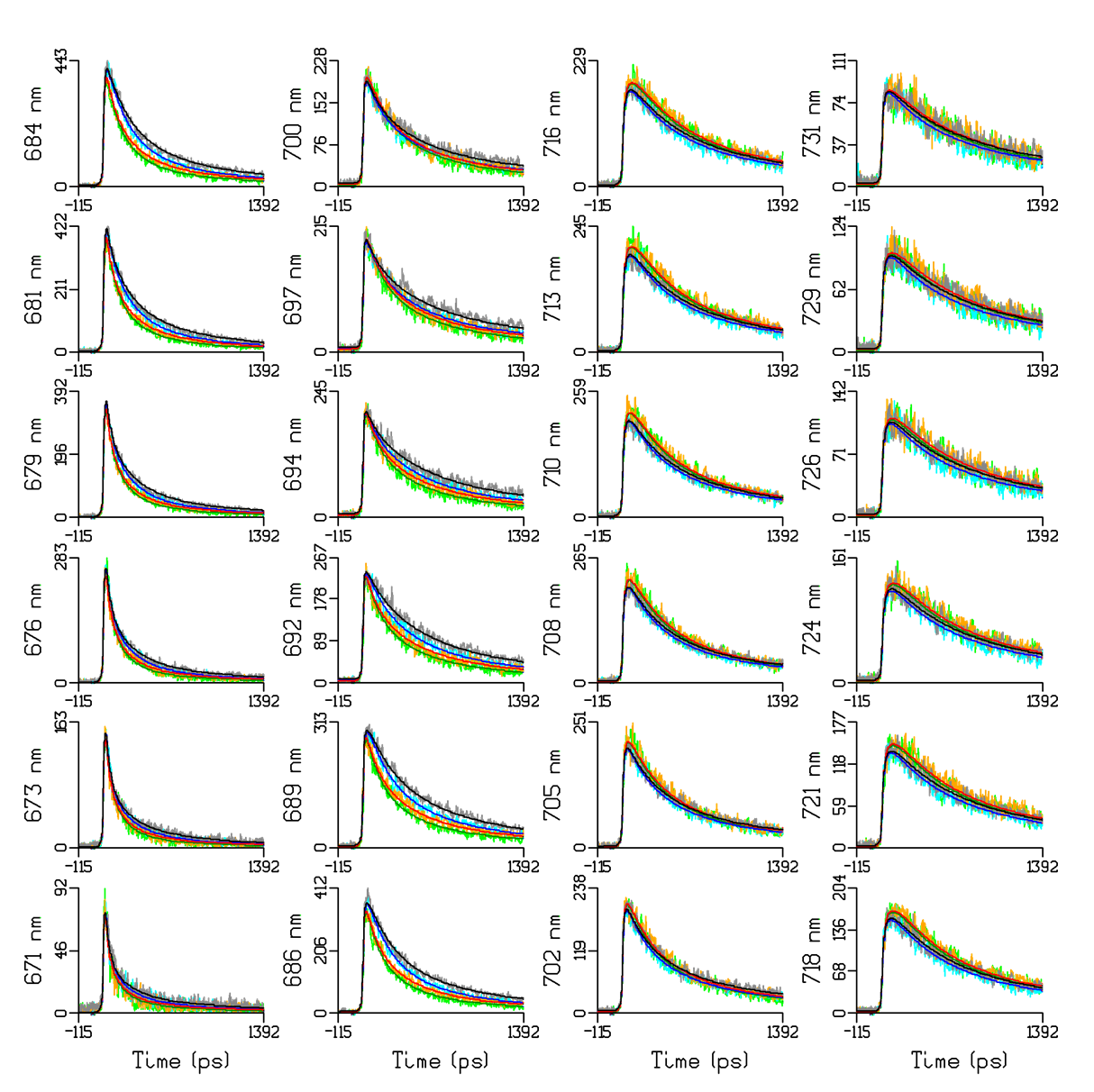


**Figure S5A** 77 K time-resolved emission decays measured at 24 wavelengths from 671 to 731 nm in *C. reinhardtii* WT cells in St1 and in St2. The cells were continuously illuminated with 400 nm laser light at repetition rate of 100 kHz. Emission decays with cumulative exposure energy of 30 mJ for St1 cells (grey) and St2 cells (orange) are shown together with their fits (respectively: black and red). Likewise, emission decays with cumulative exposure energy of 150 mJ for St1 cells (cyan) and St2 cells (light green) and their fits (respectively: blue and dark green) are indicated.


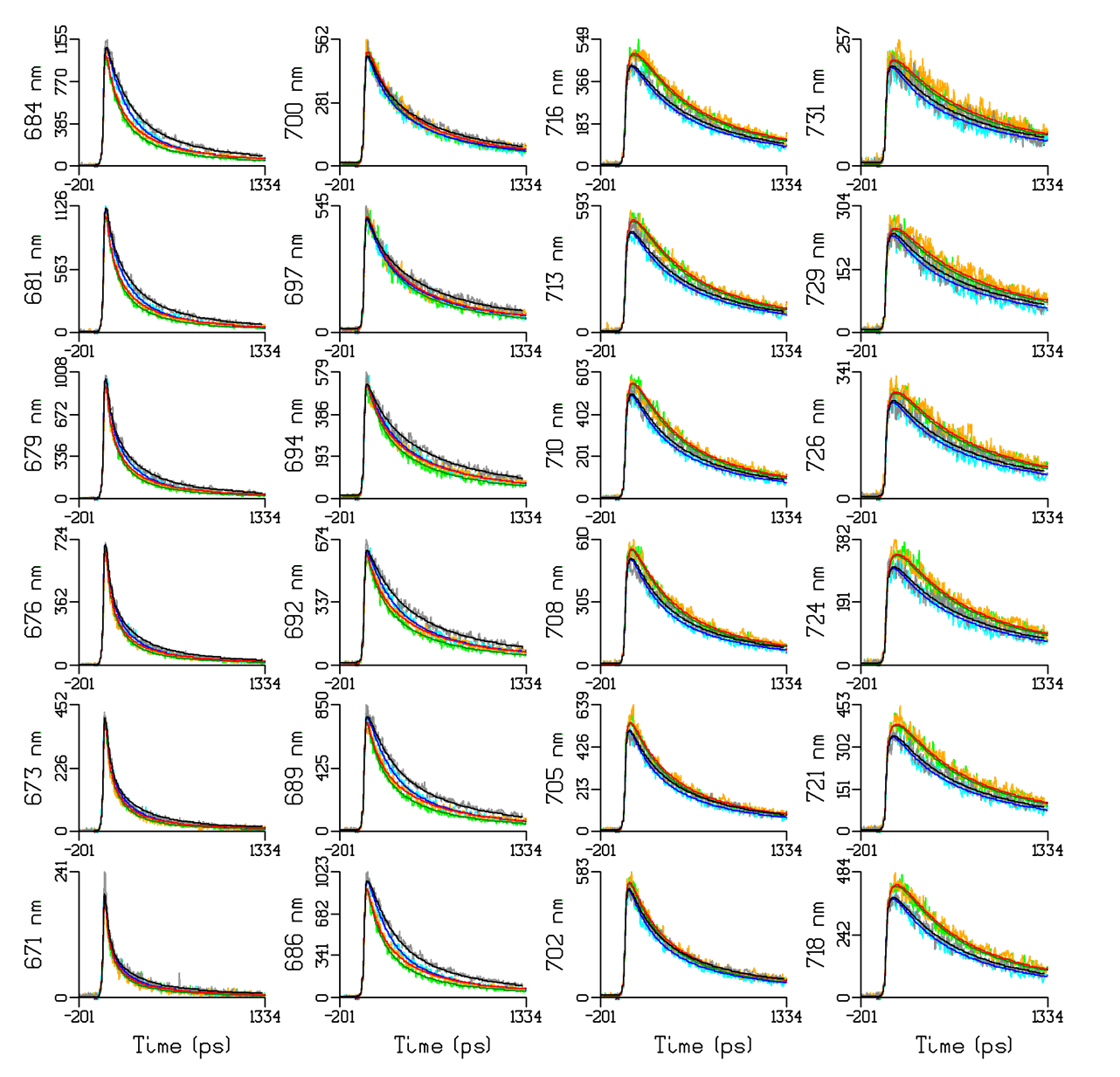


**Figure S5B** 77 K time-resolved emission decays measured at 24 wavelengths from 671 to 731 nm in *C. reinhardtii* WT cells in St1 and in St2. The cells were continuously illuminated with 400 nm laser light at repetition rate of 50 kHz. Emission decays with cumulative exposure energy of 30 mJ for St1 cells (grey) and St2 cells (orange) are shown together with their fits (respectively: black and red). Likewise, emission decays with cumulative exposure energy of 135 mJ for St1 cells (cyan) and St2 cells (light green) and their fits (respectively: blue and dark green) are indicated.


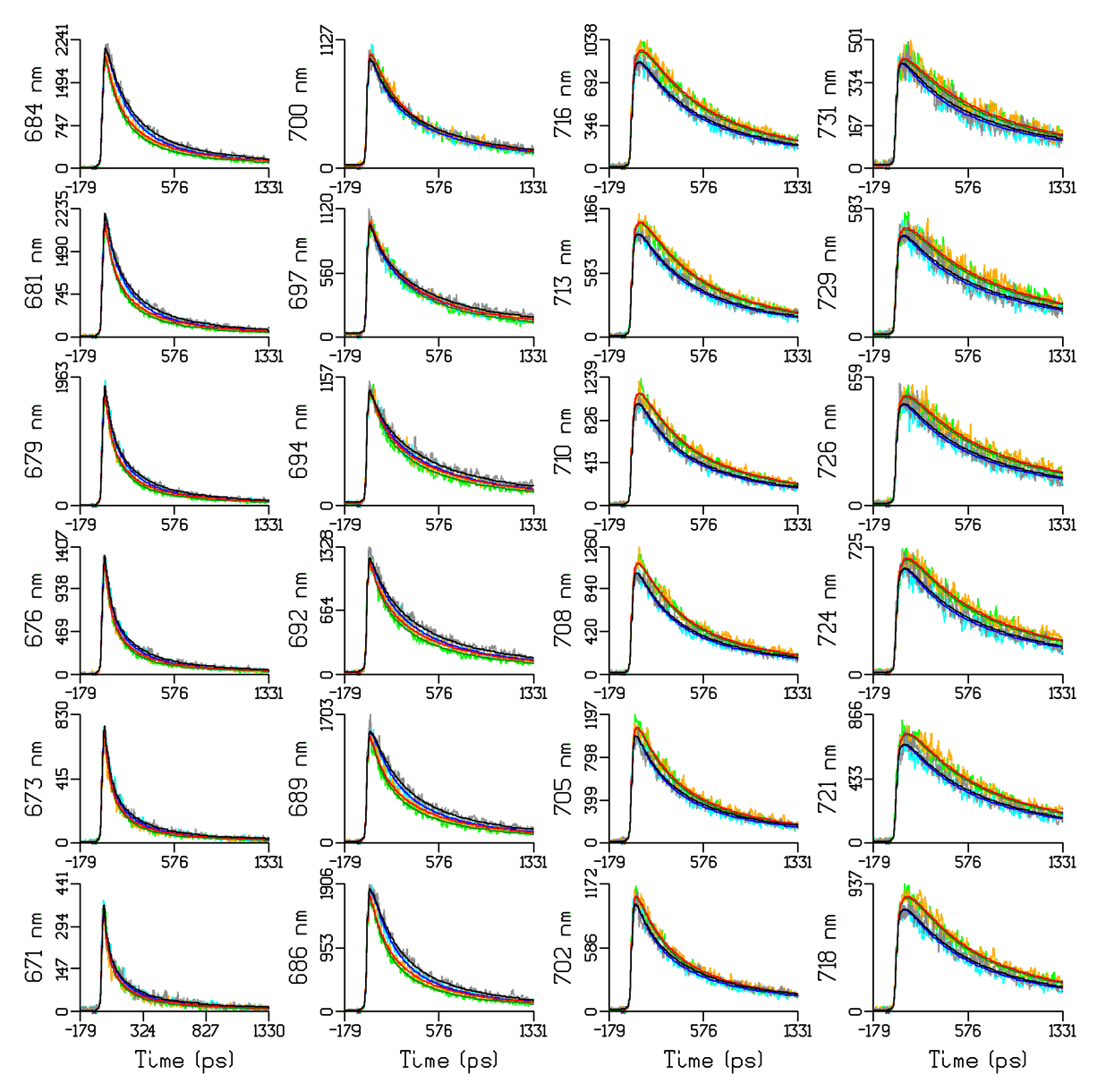


**Figure S5C** 77 K time-resolved emission decays measured at 24 wavelengths from 671 to 731 nm in *C. reinhardtii* WT cells in St1 and in St2. The cells were continuously illuminated with 400 nm laser light at repetition rate of 250 kHz. Emission decays with cumulative exposure energy of 30 mJ for St1 cells (grey) and St2 cells (orange) are shown together with their fits (respectively: black and red). Likewise, emission decays with cumulative exposure energy of 150 mJ for St1 cells (cyan) and St2 cells (light green) and their fits (respectively: blue and dark green) are indicated.


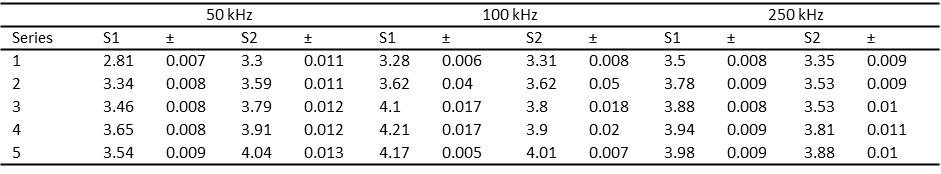


**Table S1** Estimated quenching rate constant Q and estimated standard errors of Q.


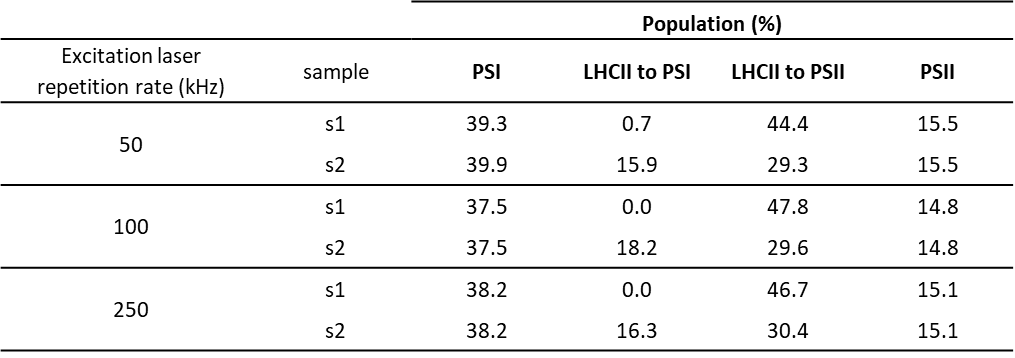


**Table S2** Population directly after 400 nm excitation per subunit of the full compartmental model depicted in Figure 4A in the main text.

## Quencher vs singlet-singlet annihilation

In the photosynthetic apparatus exposed to high excitation intensities, exciton-exciton annihilation can occur (van Amerongen et al. 2000). The resulting decrease of the excited-state lifetimes leads to lowering of the fluorescence yield. As opposed to quenching due to quencher Q, the quenching effect caused by exciton-exciton annihilation will not accumulate over times longer than tens of µs. It is however still important to assess the presence of exciton-exciton annihilation under the given measuring conditions because it can change the dynamics of the accumulation of quencher Q.

Singlet-singlet annihilation can occur in PSII supercomplex when two or more singlet excitons are present in the complex at the same time. In order to calculate the probability of such a state under the present experimental conditions, we first estimate the absorption cross-section of the PSII supercomplex taking as a reference the absorption cross-section of the LHCII trimer which at 633 nm equals 1.4 x 10^-15^ cm^2^ (Gruber et al. 2015, 2016). The PSII supercomplex of *C. reinhardtii* consists of a dimeric core, 6 LHCII trimers and 4 minor antenna (CP26 and CP29) and is roughly 10 times bigger than a single LHCII trimer (Drop et al. 2014). Moreover, PSII supercomplexes and BBY membranes absorb 3.2 times more at 400 nm than at 633 nm (Caffarri et al. 2014). Thus, at 400 nm the estimated absorption cross-section of PSII supercomplex of *C. reinhardtii* is 45 x 10^-15^ cm^2^. Taking into account the measuring conditions applied in our study the average absorption rate is then 2.21 x 10^-3^ excitations per pulse per PSII supercomplex. Based on the Poisson distribution the probability of creating ≥ 2 excitons in the PSII supercomplex is extremely low and equals 2.4 x 10^-6^. The same is concluded upon comparison with previous studies on LHCII trimers and aggregates (Barzda et al. 2001). There, with 2 x 10^-6^ excitation per pulse per LHCII trimer no singlet-singlet annihilation was observed. In the current work, due to the shorter excitation wavelength used (x 4), higher energy per pulse (x 48) and larger complexes (x 10), this number is roughly 2000 times bigger, giving 3.84 x 10^-3^ excitations per pulse per PSII supercomplex, which is very similar to 2.21 x 10^-3^ calculated above. Moreover, Barzda and colleagues observed singlet-singlet annihilation in LHCII aggregates only upon excitation with > 1.5 x 10^16^ photons/cm^2^ per pulse while the photon flux in the current study is six orders of magnitude lower – 4.9 x 10^10^ photons/cm^2^ per pulse. We conclude therefore that singlet-singlet annihilation is negligible in the present work.

## “Purity” of St1 and St2

In our current and previous studies (Figure 2; (Wlodarczyk et al. 2016)), we observed that the 77 K steady-state spectra of the St1 cells show a rather pronounced PSI emission. Only with chemical locking of St1 by kinase inhibitor staurosporine in our earlier work (Wlodarczyk et al. 2015) we observed a weaker PSI band in St1. Similar 77 K spectra were observed upon chemical locking for example with PSII inhibitor DCMU by Iwai et al. (2008). In a recent study, Fujita et al. (2018) induced ST with different light regimes using no chemical locking, and the resulting 80 K steady-state emission averaged over 10 random cells revealed a St1 fluorescence spectrum similar to the one observed in our current work. The authors noted however that not all of the cells showed strong state transitions. It is therefore possible that St1 presented in this work contains some admixture of St2. This result might also contribute to the fact that we did not observe a strong difference in the quencher build-up between St1 vs St2 cells. We anticipate that the apparent need of chemical locking to obtain the “pure” St1 or St2 indicates that such states are favourable physiologically.


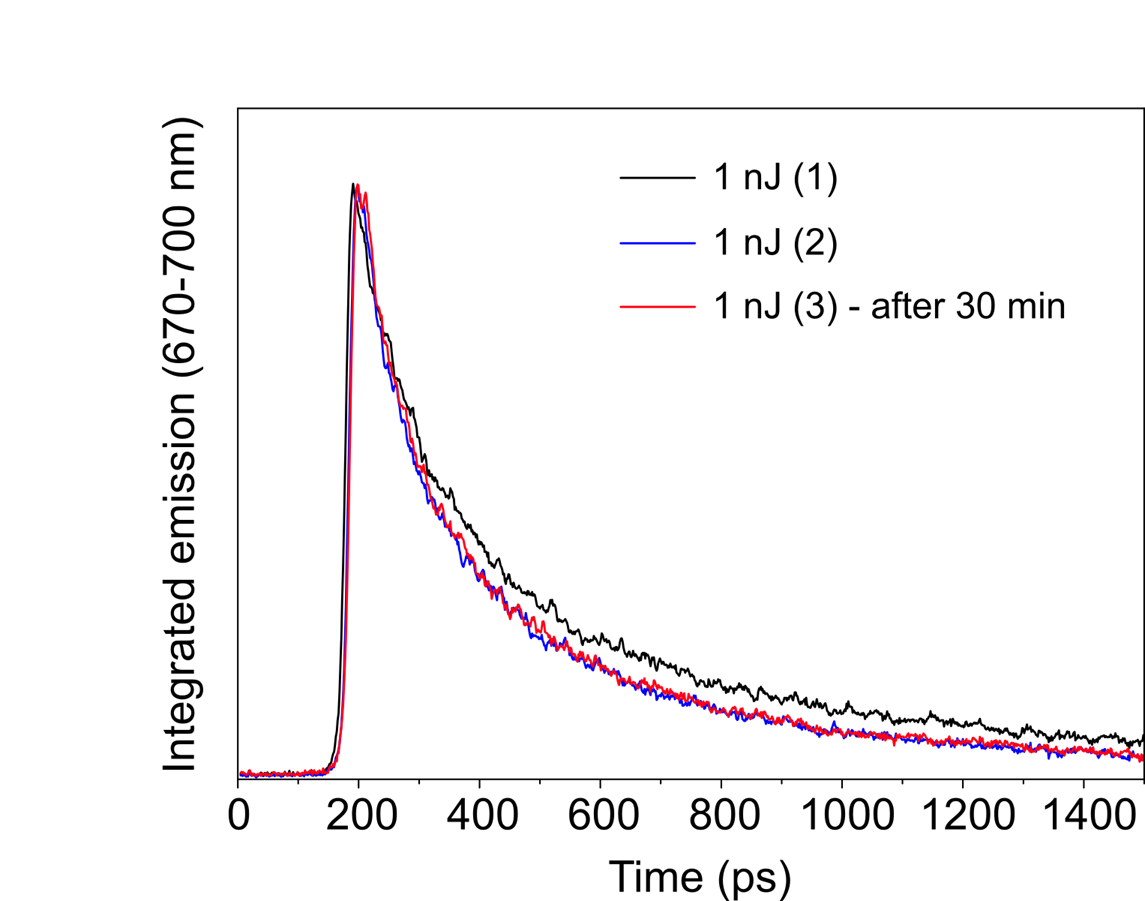


**Figure S6** Fluorescence decay of emission integrated over 670-700 nm before accumulation of the quencher (black), right after this accumulation (blue) and after subsequent 30 min of sample incubation in the dark (red). Quencher was accumulated with 10-nJ illumination at 400 nm for 2.5 min.


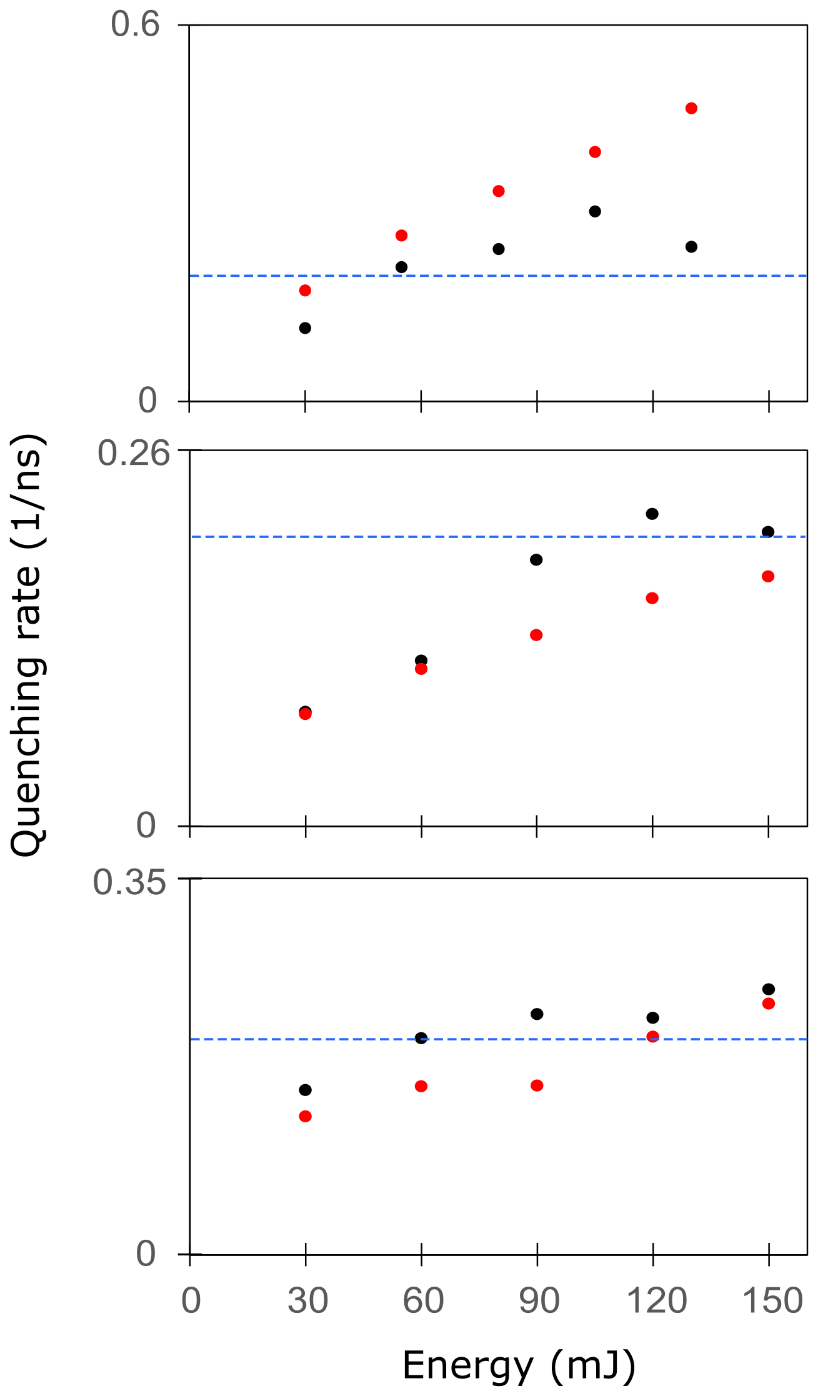


A

B

C

**Figure S7** Quenching rate constant Qred (of the red Chl compartments PSII-690 and PSII-695 in an alternative kinetic scheme, where the quencher does not reside at PSII-684) as a function of the cumulative exposure energy upon prolonged illumination *of C. reinhardtii* WT cells in St1 (black) or in St2 (red). The laser repetition rate was 50 kHz (A), 100 kHz (B) or 250 kHz (C). The decay rate constant of 0.2 ns^-1^ estimated in our previous study (Snellenburg et al. 2017) is indicated with a blue dashed line in A,B and C. Further explanation in the Discussion section.

## References

Barzda V, Gulbinas V, Kananavicius R, et al (2001) Singlet–Singlet Annihilation Kinetics in Aggregates and Trimers of LHCII. Biophys J 80:2409–2421. doi: 10.1016/S0006-3495(01)76210-8

Caffarri S, Tibiletti T, Jennings RC, Santabarbara S (2014) A comparison between plant photosystem I and photosystem II architecture and functioning. Curr Protein Pept Sci 15:296–331

Drop B, Webber-Birungi M, Yadav SKN, et al (2014) Light-harvesting complex II (LHCII) and its supramolecular organization in Chlamydomonas reinhardtii. Biochim Biophys Acta - Bioenerg 1837:63–72. doi: 10.1016/j.bbabio.2013.07.012

Fujita Y, Ito W, Washiyama K, Shibata Y (2018) Imaging of intracellular rearrangement of photosynthetic proteins in Chlamydomonas cells upon state transition. J Photochem Photobiol B Biol 185:111–116. doi: 10.1016/j.jphotobiol.2018.05.029

Gruber JM, Chmeliov J, Krüger TPJ, et al (2015) Singlet–triplet annihilation in single LHCII complexes. Phys Chem Chem Phys 17:19844–19853. doi: 10.1039/C5CP01806D

Gruber JM, Scheidelaar S, van Roon H, et al (2016) Photophysics in single light-harvesting complexes II: from micelle to native nanodisks. In: Enderlein J, Gregor I, Gryczynski ZK, et al. (eds) Single Molecule Spectroscopy and Superresolution Imaging IX

Iwai M, Takahashi Y, Minagawa J (2008) Molecular Remodeling of Photosystem II during State Transitions in Chlamydomonas reinhardtii. Plant Cell 20:2177–2189. doi: 10.1105/tpc.108.059352

Krausz E, Hughes JL, Smith PJ, et al (2005) Assignment of the low-temperature fluorescence in oxygen-evolving Photosystem II. Photosynth Res 84:193–199. doi: 10.1007/s11120-004-7078-9

Snellenburg JJ, Wlodarczyk LM, Dekker JP, et al (2017) A model for the 77 K excited state dynamics in Chlamydomonas reinhardtii in state 1 and state 2. Biochim Biophys Acta - Bioenerg 1858:64–72. doi: 10.1016/j.bbabio.2016.10.001

van Amerongen H, van Grondelle R, Valkunas L (2000) Photosynthetic Excitons. WORLD SCIENTIFIC

Wlodarczyk LM, Dinc E, Croce R, Dekker JP (2016) Excitation energy transfer in Chlamydomonas reinhardtii deficient in the PSI core or the PSII core under conditions mimicking state transitions. Biochim Biophys Acta - Bioenerg 1857:625–633. doi: 10.1016/j.bbabio.2016.03.002

Wlodarczyk LM, Snellenburg JJ, Ihalainen JA, et al (2015) Functional Rearrangement of the Light-Harvesting Antenna upon State Transitions in a Green Alga. Biophys J 108:261–271. doi: 10.1016/j.bpj.2014.11.3470
